# Optimization of the Conditions for Extraction of Serine Protease from Kesinai Plant (*Streblus asper*)Leaves Using Response Surface Methodology

**DOI:** 10.3390/molecules16119245

**Published:** 2011-11-03

**Authors:** Amid Mehrnoush, Shuhaimi Mustafa, Md. Zaidul Islam Sarker, Abdul Manap Mohd Yazid

**Affiliations:** 1 Department of Food Technology, Faculty of Food Science and Technology, Universiti Putra Malaysia, 43400 UPM Serdang, Selangor, Malaysia; Email: mehrnoush_amid@yahoo.com (A.M.); 2 Department of Microbiology, Faculty of Biotechnology and Biomolecular Science, Universiti Putra Malaysia, 43400 UPM Serdang, Selangor, Malaysia; Email: shuhaimi@biotech.upm.edu.my (S.M.); 3 Department of Pharmaceutical Technology, Faculty of Pharmacy, International Islamic University Malaysia, Kuantan Campus, Bandar Indera Mahkota, 25200 Kuantan, Pahang, Malaysia; Email: zaidul@iium.edu.my (M.Z.I.S.)

**Keywords:** extraction, enzyme, specific activity, stability, oxidizing agent

## Abstract

Response surface methodology (RSM) using a central composite design (CCD) was employed to optimize the conditions for extraction of serine protease from kesinai (*Streblus asper*)leaves. The effect of independent variables, namely temperature (42.5,47.5, *X*_1_), mixing time (2–6 min, *X*_2_), buffer content (0–80 mL, *X*_3_) and buffer pH (4.5–10.5, *X*_4_) on specific activity, storage stability, temperature and oxidizing agent stability of serine protease from kesinai leaves was investigated. The study demonstrated that use of the optimum temperature, mixing time, buffer content and buffer pH conditions protected serine protease during extraction, as demonstrated by low activity loss. It was found that the interaction effect of mixing time and buffer content improved the serine protease stability, and the buffer pH had the most significant effect on the specific activity of the enzyme. The most desirable conditions of 2.5 °C temperature, 4 min mixing time, 40 mL buffer at pH 7.5 was established for serine protease extraction from kesinai leaves.

## 1. Introduction

Peptide peptides bonds in proteins are cleaved due to the catalyzing function of the spherical structure of protease [1]. The importance of proteases lies in their versatility as agents of many critical biological processes that include the vital functioning of the regulating metabolism, gene expression, pathogenicity, modification of enzymes, and in the hydrolysis of larger proteins to short peptides for transportation and metabolism [2]. With current worldwide sales estimated at US$1 billion, industrial enzymes rank high in commercial potential [3]. Hydrolytic enzymes currently make up 75% of industrial enzymes, and proteases are among the three largest groups [4]. Plant proteases show wide substrate specificity as well as high activity and stability over a wide range of pH and temperature values and in the presence of various metal ions, inhibitors and organic solvents [5]. Such qualities make plant proteases an excellent choice for the food, medical, biotechnology and pharmacology industries [6].

Kesinai (*Streblus asper*) is a tropical tree indigenous to Malaysia, Philippines, China, and India and many other tropical and sub-tropical countries [7]. It has been reported that the different parts of the plant can be used for various applications. The bark extract, for example, has been used in treating fever, dysentery and gingivitis. The root of the plant has been known to cure ulcers, sinus problems and as an antidote for snake bites [8], while the milky juice of kesinai has also been applied to heal wounds. Apart from all these uses, kesinai leaves are a known rich source of proteases [9].

In the commercial production of enzymes, microorganisms are the primary sources. Presently, 50% of commercial enzymes are sourced from fungi and yeast and 35% from bacteria. The remaining enzyme production, however, is through extraction from plants or animal sources [10]. During enzyme extraction, clarification is applied to eliminate particulate materials from the surrounding liquid (e.g., fermentation medium or buffer). Due to the small size of bacterial cells and small differences between the density of the fermentation medium and these cells, in microbial enzyme production the clarification step is very time consuming. However, large cells such as yeast cells can be obtained by decantation but in contrast; plant residues can simply be separated by centrifugation or filtration [11]. Therefore, kesinai leaves can be utilized as a valuable and cost-effective media source for producing the natural enzyme.

There is no available literature about optimization of serine protease extraction from kesinai leaves using RSM. This study investigates the possibility of a relationship between the extraction conditions and enzymatic properties of serine protease from kesinai leaves and discusses the development of an extraction process by response surface methodology. The main objective of the study is to determine the ideal conditions for serine protease extraction that will provide maximum specific activity, storage stability, temperature and oxidizing agent stability in terms of other responses.

## 2. Results and Discussions

### 2.1. Response Surface Analysis

The results of the analysis of variance (ANOVA, which was performed with Fisher’s statistical test for fitting the model relating to the response to independent variables for each response variable, are shown in Table 1 (a–d).

**Table 1:** Analysis of variance (ANOVA) for response variables (a) Specific activity (b) Storage stability (c) Thermal stability (d) Oxidizing agent stability.

(a)					
**Source**	**SS**	**DF**	**MS**	**F-value**	**Probability > F**
Model	4062.7	12	885.31	2.41	<0.0001
Residual	1120.32	4	252.41		
Lack of fit	1041.21	3	580.75	70.04	0.0003
Pure error	8.2	3	0.71		
Total	5184.58	17			
*R^2^*: 0.989; CV = 4.30; Sum of Squares; DF: Degree of Freedom; MS: Mass Square.					
(b)					
**Source**	**SS**	**DF**	**MS**	**F-value**	**Probability > F**
Model	5360.58	9	595.62	3.87	<0.0001
Residual	1076.57	7	153.8		
Lack of fit	1070.34	4	356.78	82.98	0.0003
Pure error	6.23	3	1.56		
Total	6585.58	14			
*R^2^*: 0.999; CV = 1.48.					
(c)					
**Source**	**SS**	**DF**	**MS**	**F-value**	**Probability > F**
Model	10552.2	15	879.35	0.98	<0.0001
Residual	1602.01	9	155.71		
Lack of fit	989.98	5	482.63	215.4	0.0003
Pure error	6.23	4	0.2		
Total	11387.5	21			
*R^2^*: 0.999; CV = 0.92.					
(d)					
**Source**	**SS**	**DF**	**MS**	**F-value**	**Probability > F**
Model	8152.47	11	679.4	1.2	<0.0001
Residual	2314.41	6	171.5		
Lack of fit	1128.34	4	525.78	92.3	0.0003
Pure error	3.45	4	0.6		
Total	9102.48	17			
*R^2^*: 0.994; CV = 1.92.					

The results indicate that quadratic regression models are significant because F-test values for each response variable are less than 0.05 and the values of model F indicate that the response surface models are significant. Furthermore, the lack of fit, which determines the fitness of the model, show a significant F-value for response surface models in the ANOVA tables, therefore, ensuring a satisfactory fit between response surface models and experimental data [12]. Also, the *R^2^* values indicating the high percentage (>90%) of the response variables are explained by the model [13]. In addition, the small value of coefficient variation (CV) implies that the experiments conducted are precise and reliable [14]. The estimated regression coefficient, *R*^2^, adjusted *R*^2^, as long as *p*-value of the response surface models is given in Table 2 and Table 3. As shown in these tables, high *R*^2^ and adjusted *R*^2^ values ranging from 0.994 to 0.999 and 0.955 to 0.995 were obtained for all response variables. The coefficients of the regression equations were calculated using Minitab and the following regression equations were obtained for: (a) specific activity, (b) storage stability, (c) thermal stability and (d) oxidizing agent stability:

[a] Specific activity = 162.13+ 44.97 (mixing time)+ 9.67 (buffer content) + 34.79 (pH of buffer)+41.27 (temperature)^2^+ 7.21 (mixing time)^2^+ 22.13 (buffer content)^2^ + 8.31(pH of buffer)^2^ + 53.15 (mixing time *pH of buffer)
[b] Storage stability= 86.38 + 2.50 (mixing time) + 3.25 (buffer content) + 2.57 (pH of buffer) +13 (temperature)^2^ + 4.65 (mixing time)^2^+ 7.27 (buffer content)^2^ +23.27 (pH of buffer)^2^ +8.12 (mixing time *buffer content) +2.72 (buffer content*pH of buffer)
[c] Thermal stability = 82.36 + 3 (mixing time) +16.5 (buffer content) +2.5 (pH of buffer) +11.61 (mixing time)^2^ + 12.36 (buffer content)^2^ + 38.5 (pH of buffer)^2^ + 13.87 (temperature *mixing time) +14.25 (temperature*pH of buffer)
[d] Oxidizing agent stability = 79.15+ 5.50 (mixing time) +11.50 (buffer content) +2.57 (pH of buffer) +2.25 (temperature)^2^ + 14.25 (mixing time)^ 2^ + 14.02 (buffer content)^2^+ 7.90 (pH of buffer)^2^ +8.72 (mixing time *buffer content).

In addition, the linear, quadratic and interaction effect of each independent variable on response variables, namely, specific activity (Y_1_), storage stability (Y_2_), thermal stability (Y_3_) and oxidizing agent stability (Y_4_) are presented in Table 4. From the results shown in Table 4, the main and quadratic effects of mixing time, buffer content, pH of buffer, and the interaction effect of mixing time and buffer content show that there is significant (*p* < 0.05) effect in most of the response surface models, while the main effect of temperature indicates the least significant (*p *< 0.05) effect on all the response variables.

**Table 2:** *p*-value of each independent variable effect in polynomial response surface model.

Model term	*p*-value of each independent variable			
	Specific activity	Storage stability	Thermal stability	Oxidizing agent stability
X_1_	0.153	0.411	0.169	0.131
X_2_	0.002	0.002	0.001	0.000
X_3_	0.001	0.001	0.000	0.006
X_4_	0.000	0.001	0.000	0.001
X_1_^2^	0.014	0.020	0.327	0.044
X_2_^2^	0.001	0.001	0.000	0.000
X_3_^2^	0.008	0.000	0.002	0.000
X_4_^2^	0.000	0.001	0.000	0.017
X_1_X_2_	0.202	0.098	0.004	0.502
X_1_X_3_	0.138	0.351	0.223	0.308
X_1_X_4_	0.081	0.123	0.000	0.056
X_2_X_3_	0.102	0.001	0.134	0.000
X_2_X_4_	0.000	0.182	0.073	0.128
X_3_X_4_	0.092	0.002	0.188	0.231

X_1_, X_2_ , X_3_ and X_4_: The main effect of temperature, mixing time, buffer content and pH of buffer, respectively; X_1_^2^, X_2_^2^, X_3_^2^ and X_4_^2^: The quadratic effect of temperature, mixing time, buffer content and pH of buffer, respectively; X_1_X_2_: The interaction effect of temperature and mixing time; X_1_X_3_: The interaction effect of temperature and buffer content; X_1_X_4_: The interaction effect of temperature and pH of buffer; X_2_X_3_: The interaction effect of mixing time and buffer content; X_2_X_4_: The interaction effect of mixing time and pH of buffer; X_3_X_4_: The interaction effect of buffer content and pH of buffer.

**Table 3:** Regression coefficients, *R*^2^, adjusted *R*^2^ probability values of the response surface models.

Regression coefficient	Specific activity (Y_1, _ U/mL)	Storage stability (Y_2_, %)	Thermal stability (Y_3_, %)	Oxidizing agent stability (Y_4_, %)
b_0_	162.13	86.38	82.36	79.15
b_1_	9.12	0.57	1.00	6.25
b_2_	44.97	2.50	3.00	5.50
b_3_	9.67	3.25	16.50	11.50
b_4_	34.79	2.75	2.50	2.75
b_1_^2^	41.27	13.00	1.91	2.25
b_2_^2^	7.21	4.65	11.61	14.25
b_3_^2^	22.13	7.27	12.36	14.02
b_4_^2^	8.31	23.27	3.86	7.90
b_12_	6.71	1.83	13.87	10.23
b_13_	3.27	1.01	2.52	7.89
b_14_	9.12	11.48	14.25	13.26
b_23_	13.26	8.12	10.80	8.72
b_24_	53.15	2.68	0.97	15.61
b_34_	1.12	9.27	1.60	12.03
*R* ^2^	0.989	0.999	0.999	0.994
*R*^2^ (adj.)	0.955	0.995	0.989	0.988
Regression (*p*-value)	0.001^a^	0.000^a^	0.001^a^	0.002^a^

^bi^: The estimated regression coefficient for the main linear effects. ^bii^: The estimated regression coefficient for quadratic effects. ^bij^: The estimated regression coefficient for the interaction effects. ^1^: Temperature; ^2^: Mixing time; ^3^: buffer content; ^4^: pH of buffer. ^a^ Significant *(p* < 0.05).

**Table 4:** F-ratio and p-value for each independent variable effect in the polynomial response surface models.

Variables		Main effects				Quadratic effects				Interaction effects					
		X_1_	X_2_	X_3_	X_4_	X_1_^2^	X_2_^2^	X_3_^2^	X_4_^2^	X_1_X_2_	X_1_X_3_	X_1_X_4_	X_2_X_3_	X_2_X_4_	X_3_X_4_
**Specific activity (Y_1_, ** ** U/mg)**	p-value	0.153	0.002 ^a^	0.001 ^a^	0.000 ^a^	0.014 ^a^	0.001 ^a^	0.008 ^a^	0.000 ^a^	0.202	0.138	0.081	0.102	0.000 ^a^	0.092
	F-ratio	2.82	36.72	57.76	148.10	13.83	62.25	18.49	131.10	0.66	2.56	2.07	0.033	125.44	4.04
**Storage stability (Y_2_, %)**	p-value	0.411	0.002 ^a^	0.001 ^a^	0.001 ^a^	0.020 ^a^	0.001 ^a^	0.000 ^a^	0.001 ^a^	0.098	0.351	0.123	0.001^a^	0.182	0.002 ^a^
	F-ratio	0.82	14.06	62.09	60.84	53.58	97.41	81.90	30.25	2.56	0.08	1.96	107.32	1.69	52.85
**Thermal stability (Y_3_, %)**	p-value	0.169	0.001 ^a^	0.000 ^a^	0.000 ^a^	0.327	0.000 ^a^	0.002 ^a^	0.000 ^a^	0.004 ^a^	0.223	0.000 ^a^	0.134	0.073	0.188
	F-ratio	3.25	441.00	384.16	144.96	1.36	364.04	310.81	538.24	234.09	1.44	265.69	0.64	4.41	1.44
**Oxidizing agent stability (Y_4_, %)**	p-value	0.131 ^a^	0.000 ^a^	0.006^ a^	0.001^ a^	0.044 ^a^	0.000 ^a^	0.000 ^a^	0.017	0.502	0.308	0.056	0.000 ^a^	0.128	0.231
	F-ratio	1.44	189.00	21.16	43.03	11.28	148.59	154.00	12.39	0.04	1.08	4.96	197.96	2.40	0.022

X_1_, X_2_, X_3_ and X_4_: The main effect of temperature, mixing time, buffer content and pH of buffer, respectively. X_1_^2^, X_2_^2^ X_3_^2^ and X_4_^2^: The quadratic effect of temperature, mixing time, buffer content and pH of buffer, respectively. X_1_X_2_: The interaction effect of temperature and mixing time. X_1_X_3_: The interaction effect of temperature and buffer content. X_1_X_4_: The interaction effect of temperature and pH of buffer. X_2_X_3_: The interaction effect of mixing time and buffer content. X_2_X_4_: The interaction effect of mixing time and pH of buffer. X_3_X_4_: The interaction effect of buffer content and pH of buffer. ^a^ significant at (p < 0.05).

### 2.2. Specific Activity of Seine Protease

**Figure 1:**
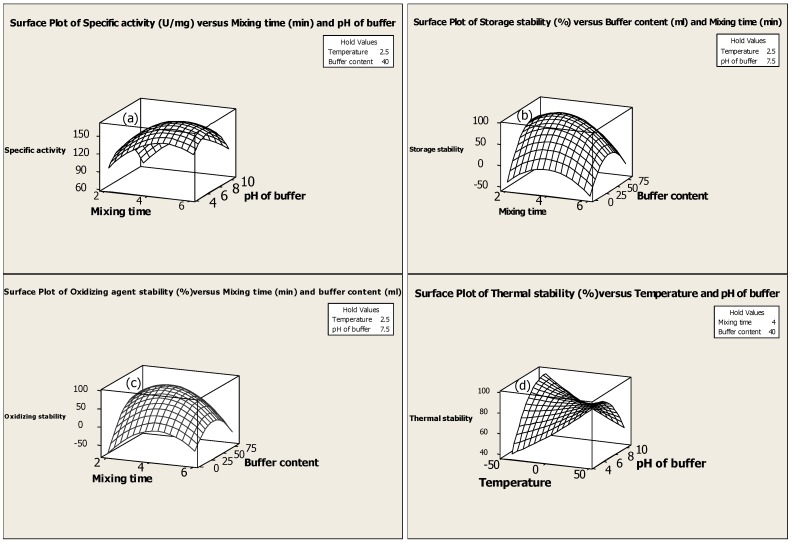
Response surface plots showing the interaction effects of (a) specific activity (b) storage stability and (c) oxidizing agent stability (d) Thermal stability.

The specific activity of serine protease was significantly (*p* < 0.05) influenced by the main effects of the mixing time, pH of buffer and buffer content, the quadratic effects of all independent variables as well as the interactive effect of mixing time and pH of buffer (Table 4). Equation 1 shows the direct relationship between the specific activity and total activity of the enzyme, thus indicating that a higher level in total activity causes a corresponding increase in the specific activity of the enzyme. From the results it can be seen that the buffer pH has the most significant (*p *< 0.05) effect on specific activity (Y_2_) of the enzyme (Table 4). In general, the highest enzyme activity is achieved when the ionisable groups of enzyme are in an appropriate ionic form and a well-maintained active site allows unrestricted access to substrates [15]. Therefore, changes in ionization of enzyme will have a negative effect on the activity of the serine protease. As shown in Figure 1(a), the highest specific activity (Y162.4 U/mg) of serine protease is obtained at pH 7.5, while the activity of the enzyme is decreased at higher and lower pH.

The mixing time is also another important parameter for the extraction of enzyme because an inappropriate time could damage the natural structure of the enzyme and cause a decline in enzyme activity. The results indicate that 4 min of mixing time is a suitable time for extraction of serine protease from kesinai leaves. The lower buffer content is not suitable for the extraction of the enzyme because the buffer content is inadequate to penetrate the solid mass. On the other hand, the higher amount of buffer would dilute the solution causing a decrease in the specific activity of the serine protease. Therefore, specific activity of serine protease is significantly increased at 40 mL of sodium phosphate buffer at pH 7.5. In addition, the desirable temperature for serine protease extraction is found to be 2.5 °C, and a higher or lower temperature will result in negative effects on total activity and lead to a decrease in specific activity.

### 2.3. Storage Stability of Serine Protease

One of the desirable outcomes of extraction optimization is the higher stability of the enzyme during storage, for according to Zhu *et al.* [16] storage stability can be measured by the total activity of the enzyme. As shown in Table 4, the interaction effects of mixing time and buffer content had the most significant effect (*p *< 0.05) on storage stability of extracted serine protease. Based on the results, storage stability of serine protease is significantly decreased at 5 min of mixing time, indicating that an inappropriate mixing time can affect the tertiary structure of the protein, thus demonstrating that inappropriate blending time can seriously damage the protein structure in the solution due to the shear forces of the Waring blender. Also, it should be noted that a shorter mixing time (>4 min) is not sufficient for extraction of serine protease from kesinai leaves, as equation 2 showed that the enzyme storage stability has direct correlation with enzyme activity thus when time of mixing is not sufficient total activity of extracted enzyme decreases as the result of decrease in storage stability was observed. The highest serine protease stability (86.8%) of the enzyme is obtained with 4 min of mixing time, which is used for the extraction.

From the results obtained, it is not experimentally feasible to achieve high total enzyme activity as well as storage stability using an excess amount of buffer. The results show that increasing the buffer content above the optimum value will dilute the solution excessively and result in reduced total enzyme activity and storage stability. The findings indicate that the optimum value of buffer content to achieve the highest storage stability is 40 mL of 100 mM sodium hydrogen phosphate at pH 7.5. As shown in Figure 1(b), when the buffer content is increased from 0 to 40 mL, the storage stability is increased but an increase of the buffer content from 40 to 60 mL, results in significantly reduced storage stability. As such, the amount of buffer is one of the crucial factors to be carefully considered in enzyme extraction.

### 2.4. Thermal Stability of Serine Protease

**Figure 2:**
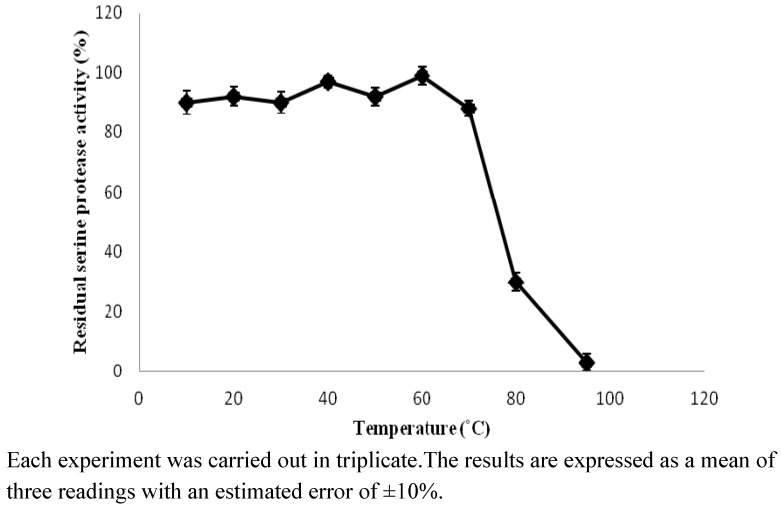
The influence of temperature on stability of kesinai serine protease.

As mentioned earlier, the thermostability of serine protease was investigated by measuring the residual enzyme activity after incubation at different temperatures from 10 to 95 °C. The results indicated that there was retention of more than 85% activity when the protease was incubated between 10 °C and 70 °C for 1h. Figure 2 shows that any temperature from 75 °C and higher sharply decreases the activity of the extracted serine protease. The molecular structure of the enzyme is an important factor for enzyme stability in relation to temperature. It appears that the structure of the enzyme is compact, thus this enzyme is stronger when the temperature rises. Moreover, statistical prediction indicates that the enzyme is thermostable because the main and quadratic effects of temperature do not show any significant (*p* < 0.05) effect on thermal stability of kesinai serine protease (Table 4). Thermal stability is desirable because the efficiency of enzymes is markedly enhanced at higher temperatures. Holding time for specific processes can be shortened, thus minimizing undesirable chemical reactions. This is most important in food processing, during which nutrients may be lost during processing at high temperatures. In addition, conducting thermal processes at higher temperatures reduces microbial contamination [17], and the significant (*p* < 0.05) interaction effect of temperature and pH of buffer is in Figure 1(d).

### 2.5. Oxidizing Agent Stability of Serine Protease

The main effects of mixing time, buffer content, pH of buffer and the quadratic effects of temperature, mixing time, buffer content and pH of buffer, as well as the interaction effects of mixing time and buffer content, had significant (*p *< 0.05) effects on the stability of serine protease from kesinai leaves towards oxidizing agents (Table 4). To be a vital component in detergents, for example, a protease must show a high level of compatibility and stability with oxidizing agents which are commonly used in detergent compounds. Therefore, the stability of kesinai serine protease was determined in the presence of H_2_O_2_ as one of the most common oxidizing agents used in the detergent industry [18]. Based on the results, 79.3% of serine protease stability was retained after incubation of the enzyme for 1 h at 60 °C in the presence of 5% (v/v) hydrogen peroxide. The high oxidizing agent stability of serine protease was achieved using optimization extraction which provided suitable formation of the enzyme with unrestricted access to substrates.

From the optimization results, the highest oxidizing agent stability of the enzyme is predicted to be at a combined level of 2.5 °C temperature, 4 min mixing time, and 40 mL sodium hydrogen buffer at pH 7.5. Also, the results show that the interaction of mixing time and buffer content has the most significant (*p* < 0.05) effect on serine protease oxidizing agent stability (Table 4). Figure 1(c) shows how the oxidizing agent stability of serine protease is enhanced when the amount of buffer is increased to 40 mL and mixing time is 4 min. This indicates that using an optimum mixing time of extraction helps to maintain the serine protease structure with unhindered access of the substrate to the active site. Additionally, this amount of buffer has sufficient capacity for extraction of kesinai serine protease and the enzyme, therefore, is oxidizing agent stable when employing optimized extraction.

### 2.6. Optimization Procedure

The target of optimization is to achieve the best levels of independent variables that result in desirable response goals so the individual and overall optimization procedures were performed to achieve this target. To determine the optimum region, numerical and graphical optimization procedures were employed. Graphical optimization (using 3D surface plot) was employed to determine the optimum region, thus in order to better understand the interaction effect of the graphed independent variables on response variables, graphic 3D surface plots were used to illustrate the reduced response models. The 3D plots were generated within the experimental range by fixing and varying a centre point and two variables. With the aid of the response optimizer, a numerical optimization was performed to determine the exact optimal levels of individual and simultaneous multiple response optimizations in order to achieve the desired response goals. Furthermore, in order to determine the adequacy of the response surface equations, a comparison was made between the experimental data and predicted values from the reduced response regression [19]. The results indicated that the extraction using 40 mL buffer at pH 7.5 at 2.5 °C for 4 min mixing time provided the overall optimum region in terms of all serine protease properties.

### 2.7. Model Validation

**Figure 3:**
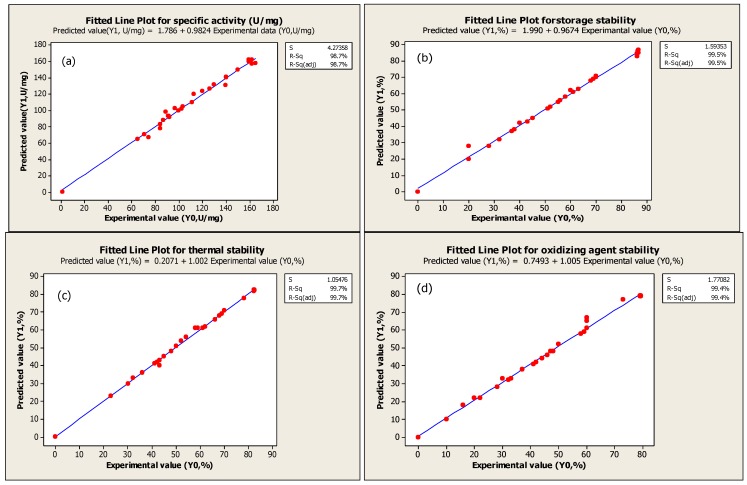
Fitted line plot indicating the closeness between predicted values (Y1) and experimental values (Y0) for specific activity (a), storage stability (b) thermal stability (c), and oxidizing agent stability of serine protease (d).

The adequacy of the response surface equations is indicated by a comparison between the experimental value and the predicted data [20]. The comparison is done by generating a fitted-line plot (with experimental values on X-axis and predicted values on Y-axis) for the results obtained, showing how close it is to or how far it deviates from the fitted line. Figure 3a–d show the overall closeness of these variables, thus indicating that the response surface model is adequate for predicting the varied enzymatic properties as functions of the conditions in extraction. Thus, based on the result, the optimum points for temperature, mixing time, buffer content and pH of buffer are 2.5 °C, 4 min, 40 mL at pH 7.5, respectively.

## 3. Experimental

### 3.1. Materials

The kesinai (*Streblus asper*) leaves were obtained from several kesinai plants available at Universiti Putra Malaysia, (Selangor, Malaysia) Science Park. All chemicals and reagents used were analytical grade. Bovine serum albumin (BSA), azocasein and Bradford reagent were supplied by Sigma Chemical Co., (St. Louis, MO, USA). Polyethylene glycol (PEG), trichloroacetic acid (TCA, 99%), di-sodium hydrogen anhydrous, sodium hydrogen phosphate monohydrate, di-potassium hydrogen phosphate and potassium di-hydrogen phosphate, were purchased from Merck (Darmstadt, Germany).

### 3.2. Serine Protease Extraction

Twenty grams of fresh kesinai leaves were washed with double-distilled water and then blended with 0–80 mL of 100 mM sodium phosphate hydrogen at pH 4.5–10.5 by using a Waring commercial laboratory blender 32BL79 (Torrington, CT, USA) for 2–6 min at high speed and different temperatures (−42.5 to 47.7 °C) (see Table 5). The homogenate was produced by filtration of the crude enzyme sample through a two-layer cheesecloth filter. The filtrate was cooled to 4 °C and centrifuged (Sartorius Model 3–18 k, Sartorius AG, Weender Land Strasse, Gottingen, Germany) at 8,000 rpm for 20 min at 4 °C, after which the supernatant was collected to determine the serine protease activity and protein concentration.

**Table 5:** Level of independent variables established according to central composite design (CCD).

Independent variables	Independent variables level				
	Low	Center	High	Axial (−α)	Axial (+α)
Temperature (°C)	−20	2.5	+25	−42.5	47.5
Time (min)	3	4	5	2	6
Buffer content (mL)	20	40	60	0	80
pH of buffer	6	7.5	9	4.5	10.5

### 3.3. Determination of Serine Protease Activity

Protease activity of semi-purified enzyme was estimated according to the method described by Whooley *et al.* [21]. The proteolytic reaction mixture contained enzyme solution (0.1 mL) and 0.2% (w/v) azo-casein prepared in 100 mM sodium phosphate hydrogen (pH 7.5) buffer (1 mL). The mixture was incubated in a water-bath at 60 °C for 20 min and 30% (w/w) TCA (0.5 mL) was added to stop the reaction. The supernatant was obtained by centrifugation (13,400 rpm, for 10 min) and then filtered through a 0.22 µm film. A spectrophotometer (BioMate^TM^-3, Thermo Scientific, Alpha Numerix, Webster, NY, USA) at 335 nm was used in the determination of protease activity. The results are expressed as a mean of three readings with an estimated error of ±10%.

### 3.4. Determination of Protein Concentration

The protein contents of samples were determined using dye binding method as described by Bradford [22] and the BSA was used as standard.

### 3.5. Determination of Serine Protease Specific Activity

The specific activity of the enzyme was determined by taking the ratio of total activity of serine protease and dividing it by total protein of serine protease [23]:

Specific activity (U/mg) = Total activity (U)/Total protein (mg) (1)

### 3.6. Determination of Serine Protease Storage Stability

The extracted serine protease was stored for one week at a temperature of 4 °C and the storage stability of the enzyme was measured by the ratio of serine protease activity after storage, to its initial activity and then multiplying the result by 100 (Equation 2) [24]:

Storage stability 100% = (Enzyme activity after storage/Initial enzyme activity) × 100 (2)

### 3.7. Determination of Serine Protease Thermal Stability

Protease thermostability was also studied by incubating it in a 100 mM sodium phosphate buffer (pH 7.5) within a temperature range of 10 to 95 °C. At different intervals of 2, 5, 10, 20, 30, 40, 60 min, samples were removed and the residual proteolytic activity was determined by azo-casein at pH 7.5 and 60 °C, respectively [25].

### 3.8. Determination of Oxidizing Agent Stability

The effect of the oxidizing agent on protease activity was investigated using H_2_O_2 _at a concentration of 5% (v/v). The samples with the presence of H_2_O_2_ were incubated for 1 h at 60 °C and the residual protease activity was then tested with azo-casein as previously described [26].

### 3.9. Statistical Design

Response surface methodology was employed to establish the optimum conditions for extracting serine protease from kesinai leaves. The effect of four independent variables, namely, temperature (−42.5 to +47.5 °C), mixing time (2–6 min), buffer content (0–80 mL) and pH of buffer (4.5–10.5) on specific activity, storage stability, temperature stability, and oxidizing agent stability was investigated using a four factorial central composite design. Consequently, thirty supernatants were extracted from kesinai leaves, based on CCD, including sixteen factorial points, eight axial points (±α) and six center points (Table 5 and Table 6). There was a six-time repetition of the center point to determine the possibility of pure error [27]. The polynomial regression equation presented below was applied to investigate response surface behavior to achieve the response equation (Y).The RSM model is explained below (Equation3):

Y= β_0 _+ Σ β_i _x_i_ +Σ β_ii _x_i_^2^ +Σ β_i j _x_i _x_j_ (3)

The equation above shows that response Y is computed by the model, while *β_0_* is a constant; the linear, squared and interaction coefficients are *β_i_*_, _*β_ii_* and *β_ij_* respectively. The adequacy of the models was determined using analysis of models, lack of fit test and determination of coefficient (*R^2^*) analysis as described by Lee *et al.* [28] Meanwhile, *R^2^* of at least 0.80 is proof of a good model fit [29] while larger values of absolute t-value and smaller values of *p*-value indicate that the variables will be more significant (p < 0.05). Furthermore, in order to determine the adequacy of the response surface equations, a comparison was made between the experimental data and predicted values from the reduced response regression [20]. The design matrix of the experiment, the analysis of the data and the optimizing procedure were all done using the Minitab v.14 statistical package (Minitab Inc., State college, PA, USA).

## 4. Conclusions

In this study, RSM was used to investigate the main and interaction effects of important independent variables for extraction of serine protease from kesinai leaves. The main and quadratic effects of mixing time, buffer content and pH of buffer had significant (*p *< 0.05) effect on all response variables, thus these independent variables should be kept in all reduced response surface models.

The main and quadratic effects of temperature did not show any significant (*p* <0.05) effect on thermal stability of the enzyme and thus this enzyme is thermostable. The results indicated that the optimum conditions for serine protease extraction from kesinai leaves was achieved using 2.5 °C of temperature, 4 min of mixing time, and 40 mL of buffer content at pH of 7.5. The optimized conditions caused an increase in specific activity and stability of the serine protease from kesinai leaves. However, enzyme losses could be due to undesirable extraction methods, which may alter the natural morphology of the enzyme. It can be inferred from this present study that the release of specific activity and stability of enzyme can be controlled by appropriate proportions of the extraction variables. In addition, the successful extraction of serine protease from kesinai leaves provides potential commercial possibilities for the industrial extraction of serine protease, from an economic as well as an environmental point of view.

**Table 6:** Matrix of central composite design (CCD).

Treatments	Blocks	Temperature (X_1_, °C)	Mixing time (X_2_, min)	Buffer content (X_3_, mL)	pH of buffer (X_4_)	Specific activity (U/mL)	Storage stability (%)	Thermal stability (%)	Oxidizing agent stability
1 ^C^	1	2.5	4	40	7.5	162.4	86.8	82.6	79.3
2	1	25	3	20	6.0	161.8	80.4	73.4	62.7
3	1	25	5	60	6.0	150.0	20.0	30.0	30.6
4	1	25	3	60	9.0	130.7	52.0	62.0	43.2
5	1	−20	3	60	6.0	120.4	56.7	53.3	61.1
6	1	25	5	20	9.0	140.5	15.3	21.2	29.3
7	1	−20	3	20	9.0	10.0	73.4	66.1	52.2
8 ^C^	1	2.5	4	40	7.5	158.9	80.1	81.1	77.7
9	1	−20	5	60	9.0	110.0	58.8	38.8	33.1
10	1	−20	5	20	6.0	62.4	86.4	42.0	32.0
11 ^C^	2	2.5	4	40	7.5	162.4	85.4	80.6	79.4
12	2	25	3	60	6.0	105.3	42.7	68.0	42.1
13 ^C^	2	2.5	4	40	7.5	160.0	86.0	82.3	71.3
14	2	25	5	60	9.0	67.2	32.4	48.0	42.2
15	2	25	3	20	9.0	88.1	44.2	78.2	61.3
16	2	−20	5	60	6.0	93.8	85.8	32.2	30.3
17	2	−20	5	20	9.0	54.9	34.8	42.2	44.7
18	2	25	5	20	6.0	90.1	14.2	18.8	21.3
19	2	−20	3	60	9.0	123.0	51.0	63.2	55.8
20	2	−20	3	20	6.0	132.1	42.8	34.1	23.2
21	3	2.5	6	40	7.5	111.9	68.0	71.2	70.2
22	3	2.5	4	0	7.5	74.3	73.4	57.1	68.8
23	3	2.5	2	40	7.5	121.2	54.5	73.6	40.2
24	3	−42.5	4	40	7.5	81.4	86.1	29.2	33.4
25	3	2.5	4	40	10.5	122.2	77.0	66.6	51.7
26 ^C^	3	2.5	4	40	7.5	161.9	72.3	80.8	78.7
27	3	2.5	4	40	4.5	101.1	42.2	52.4	54.3
28 ^C^	3	2.5	4	40	7.5	162.1	86.5	81.2	79.1
29	3	2.5	4	80	7.5	115.0	69.4	53.8	56.5
30	3	47.5	4	40	7.5	51.3	23.4	38.1	22.0

^C^ center points.
